# Dietary patterns in relation to testosterone levels and severity of impaired kidney function among middle-aged and elderly men in Taiwan: a cross-sectional study

**DOI:** 10.1186/s12937-019-0467-x

**Published:** 2019-07-27

**Authors:** Adi Lukas Kurniawan, Chien-Yeh Hsu, Hsiao-Hsien Rau, Li-Yin Lin, Jane C.-J. Chao

**Affiliations:** 1 School of Nutrition and Health Sciences, College of Nutrition, Taipei Medical University, 250 Wu-Hsing Street, Taipei, 110 Taiwan; 2 Department of Information Management, National Taipei University of Nursing and Health Sciences, 365 Ming-Te Road, Peitou District, Taipei, 112 Taiwan; 3 Master Program in Global Health and Development, College of Public Health, Taipei Medical University, 250 Wu-Hsing Street, Taipei, 110 Taiwan; 4 Joint Commission of Taiwan, 31 Sec.2 Sanmin Road, Banqiao District, New Taipei City, 220 Taiwan; 5 Nutrition Research Center, Taipei Medical University Hospital, 252 Wu-Hsing Street, Taipei, 110 Taiwan

**Keywords:** Dietary pattern, Testosterone, Kidney function, Principal component analysis

## Abstract

**Background:**

Chronic Kidney Disease (CKD), characterized by an impaired kidney function, is associated with low testosterone levels. This study investigated the association between dietary patterns, testosterone levels, and severity of impaired kidney function among middle-aged and elderly men.

**Methods:**

This cross-sectional study used the database from a private health-screening institute in Taiwan between 2008 and 2010. Men aged 40 years old and older (*n* = 21,376) with estimated glomerular filtration rate (eGFR) < 90 mL/min/1.73 m^2^ and proteinuria were selected. Among 21,376 men, 256 men had available measurements of testosterone levels. Dietary assessment was conducted using a food frequency questionnaire and three dietary patterns (fried-processed, vege-seafood, and dairy-grain dietary patterns) were identified using principal component analysis.

**Results:**

Men in the lower tertiles (T1 and T2) of eGFR had significantly decreased testosterone levels by 0.8 (95% CI: − 1.40, − 0.20) and 0.9 nmol/L (95% CI: − 1.43, − 0.33). Furthermore, serum triglycerides (TG) levels were inversely associated with testosterone levels (β = − 0.51, 95% CI: − 0.77, − 0.24). Men in the higher tertile of fried-processed dietary pattern scores were associated with decreased testosterone levels by 0.8 nmol/L (95% CI: − 1.40, − 0.16), reduced testosterone-to-TG (T/TG) ratio by 1.8 units (95% CI: − 2.99, − 0.53), and increased risk of moderate/severe impaired kidney function (eGFR < 60 mL/min/1.73 m^2^) and proteinuria severity by 1.35 (95% CI: 1.15, 1.58) and 1.18 (95% CI: 1.02, 1.37) times respectively. In contrast, the vege-seafood dietary pattern was negatively associated with severity of impaired kidney function and proteinuria after multivariable adjustment, but had no association with testosterone levels and T/TG ratio.

**Conclusions:**

The fried-processed dietary pattern is negatively associated with testosterone levels but positively associated with the severity of impaired kidney function. However, the vege-seafood and dairy-grain dietary patterns appear to have beneficial effects.

**Electronic supplementary material:**

The online version of this article (10.1186/s12937-019-0467-x) contains supplementary material, which is available to authorized users.

## Background

Chronic kidney disease (CKD), characterized by an impaired kidney function, is recognized as a global health problem with major adverse outcomes that include cardiovascular disease (CVD) and end-stage renal disease (ESRD). In Taiwan, a large prospective 13-year cohort study showed that the national prevalence of CKD in 2007 was around 12% and less than 4% of the participants were aware of this disease [1]. The prevalence of CKD started to increase at age 40–44 years old (7.4%) and doubled between the ages of 55–59 years (13.1%) and 60–64 years (26.1%) [1]. Moreover, according to the US Renal Data System in 2014, Taiwan had the highest prevalence and incidence rate of ESRD worldwide, especially in the middle-aged and elderly populations [2]. Patients with ESRD spent approximately 7% of the total annual budget from the Taiwan national health insurance program, indicating that CKD not only threatens population health but also becomes a financial burden to the nation [3].

Decreased testosterone levels are the most common gonadal problem among men with impaired kidney function [4]. A cross-sectional study by Yilmaz et al. showed that serum total testosterone levels were decreased by 10% in CKD stage 2 and 42% in CKD stage 5, compared to those in stage 1 and the prevalence of hypogonadism was increased from 75% in men with CKD stage 1 to 92% in CKD stage 5 [5]. Additionally, testosterone deficiency has been associated with reduced kidney function, CKD progression, and all-cause mortality, especially by CVD. A possible explanation is that testosterone has vasodilatory effects on blood vessels, which can prevent the progression of atherosclerosis, ischemia, and endothelial dysfunction [6, 7].

Dietary patterns have been linked to many risks for developing chronic diseases [8]. In previous studies, high intake of protein and Western diet (red and processed meats, animal/saturated fats, and sugar/sweets) were associated with increased risk of CKD, while high intake of vegetables, fruits, and whole grains reduced the risk of decreased kidney function [9–11]. However, previous studies examining the effects of dietary patterns in subjects with impaired kidney function were mostly conducted in Western countries and among women [9–11]. In fact, Taiwanese men have a higher rate of kidney disease than women [1] and there is no published study investigating the correlation of dietary patterns with the severity of impaired kidney function among Taiwanese men. Although a recent study among type 2 diabetes patients in Taiwan showed that diets rich in fish and vegetables were associated with better kidney function [12], further study is still needed to investigate the association between dietary patterns and the onset of impaired kidney function in a larger sample size. Additionally, to our knowledge, few studies have described the relationship between diets and testosterone levels [13–15]. Notably, a previous study found a significant increase in serum testosterone levels in overweight or obese men on a low-fat diet for 12 weeks and weight maintenance for another 40 weeks [13]. In contrast, two small cross-sectional studies did not find an association between vegetarian diets and testosterone levels in men [14, 15]. In addition, no study has yet examined the relationship between dietary patterns and testosterone levels among men with impaired kidney function. Therefore, this study aims to investigate the relationship between dietary patterns, testosterone, and severity of impaired kidney function among middle-aged and elderly men in Taiwan.

## Methods

### Data source and study subjects

**Fig. 1:**
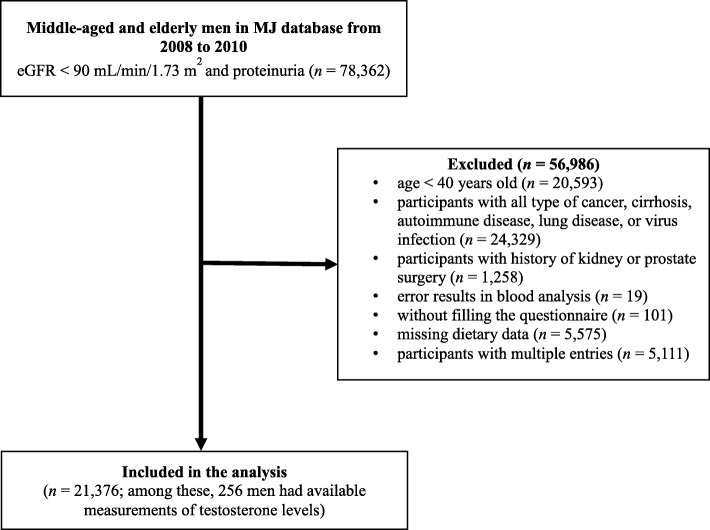
Flow chart diagram of subjects included in the study

This cross-sectional study was conducted with samples collected from Mei Jau (MJ) Health Management Institution database, Taiwan. The MJ Health Management Institution is a membership-oriented private institute with four health check-up centers (Taipei, Taoyuan, Taichung, and Kaohsiung) in Taiwan, which provides periodic health examination (mostly one examination per year per person) to its members. Subjects completed a self-reported questionnaire to collect information on sociodemographic data, lifestyle, medical history, and dietary habit before blood and urine tests, anthropometric measurement, and a physical examination. All individuals had signed a consent form authorized by the MJ Health Screening Center to process the data generated from medical screening. The data were treated as highly confidential, without personal identification, and for research purposes only. The data consisted of 78,362 middle-aged and elderly men with estimated glomerular filtration rates (eGFR) < 90 mL/min/1.73 m^2^ and proteinuria were retrieved from the MJ database between 2008 and 2010. After excluding with some criteria in Fig. 1, a total of 21,376 men were recruited for analysis. The Institutional Ethical Review of Taipei Medical University (TMU-JIRB N201802006) approved this study.

### Clinical and biochemical data

Body weight and height were measured by an auto-anthropometer (Nakamura KN-5000A, Nakamura, Tokyo, Japan). Waist and hip circumferences were measured by a flexible tape. After resting for 5 min, blood pressure was measured twice at 10 min intervals on the right arm in a sitting position by a standardized sphygmomanometer. Hypertension was defined as systolic blood pressure ≥ 140 mmHg or diastolic blood pressure ≥ 90 mmHg, use of an anti-hypertensive drug, or a self-report of hypertension diagnosis by a physician [16]. Body mass index (BMI) was calculated as weight in kilograms divided by the square of height in meters.

Fasting blood glucose (FBG), triglycerides (TG), total cholesterol (TC), high density lipoprotein-cholesterol (HDL-C), low density lipoprotein-cholesterol (LDL-C), C-reactive protein (CRP), and blood urea nitrogen (BUN) were analyzed (Toshiba C8000 auto-analyzers, Tokyo, Japan) in each subject after fasting for at least 8 h. Diabetes mellitus was defined as FBG ≥ 7.0 mmol/L (126 mg/dL), use of a hypoglycemic agent, or a self-report of diabetes diagnosis by a physician [17]. Serum testosterone levels were measured by chemiluminescent microparticle immunoassay (CMIA, Architect System, Abbott, IL, USA). The architect testosterone assay measures total testosterone. However, among 21,376 men, only 256 men had serum testosterone data. Serum creatinine levels were analyzed by uncompensated Jaffe method with alkaline picrate kinetic test. The Roche Miditron M semi-automated computer-assisted urinalysis system (Combur-10 test M dipstick, Basel, Switzerland) was used to measure urinary protein. The result was reported as one plus (equivalent to micro-albuminuria) or more pluses (equivalent to macro-albuminuria). All specimens were analyzed at MJ Health Screening Central Laboratory. The internal and external quality control techniques were performed by the laboratory and the calibrations were supplied by the manufacturer [18]. The laboratory followed Westgard multi-rules for sample quality control and the coefficient of variation ranged from 1 to 3%. Furthermore, we used the equation from Chronic Kidney Disease Epidemiology Collaboration (CKD-EPI) to estimate eGFR [19]:

If creatinine ≤79.56 μmol/L (0.9 mg/dL) in male:

$$ \mathrm{eGFR}\ \left(\mathrm{mL}/\min /1.73\ {\mathrm{m}}^2\right)=141\times {\left(\mathrm{creatinine}/0.9\right)}^{-0.411}\times {(0.993)}^{\mathrm{age}} $$

If creatinine > 79.56 μmol/L (0.9 mg/dL) in male:

$$ \mathrm{eGFR}\ \left(\mathrm{mL}/\min /1.73\ {\mathrm{m}}^2\right)=141\times {\left(\mathrm{creatinine}/0.9\right)}^{-1.209}\times {(0.993)}^{\mathrm{age}} $$

Based on eGFR levels, we categorized men with impaired kidney function into mild (*n* = 19,519) and moderate/severe (*n* = 1,857) groups which were defined as eGFR 60–89 mL/min/1.73 m^2^ with positive urinary protein and eGFR < 60 mL/min/1.73 m^2^, respectively [20].

### Dietary assessment and other covariates

A standardized and validated self-administered semi-quantitative food frequency questionnaire was used to assess dietary habit [21, 22]. The frequency and servings of dietary intake were assessed according to the consumption of twenty-two food groups daily or weekly in the past month before data collection. The frequency intake for each food group was categorized into five responses from the lowest to the highest using the following units of measurement: a bowl for vegetables and oil added vegetables or salad, a glass for milk and sugar-sweetened beverages, and a serving for the remaining food groups. The definition of the portion size (serving) was described previously [22].

The demographic and lifestyle data such as age, education levels, marital status, family income, smoking, drinking alcohol (no, < 1 time/week; yes, ≥ 1–2 times/week), sleeping, physical activity, and medical history (having a history and using drug for CVD, hypertension, and diabetes mellitus) were also collected. The questions regarding physical activity were the frequency and time for exercise during the past 2 weeks. The frequency of exercise had five response options that ranged from none or rarely to 2–3 times per day, while the exercise time had four response options that ranged from less than 30 min to more than 2 h. We defined physical activity as ‘yes’ if the subject engaged in exercise for more than 1 h in a week and ‘no’ if otherwise [22]. A structured questionnaire used to collect demographic and lifestyle data in this study was validated prior to its use and has been documented elsewhere [1, 18]. Moreover, CVD was defined as having a history of CVD or use of a cardiovascular drug.

### Statistical analysis

Statistical analysis was performed by using IBM® SPSS Statistics version 20.0 (International Business Machines Corp., Armonk, NY, USA). Categorical and continuous variables were presented as number (percentage) of the subjects and mean ± standard deviation (SD), respectively. A general linear model and a chi-square test for continuous and categorical data respectively, were used for analyzing the characteristics across tertiles of eGFR. Due to the limited number of men who had testosterone levels, we decided to use tertiles of eGFR for the characteristics of the study instead of impaired kidney function categories (only few men who had testosterone levels in the moderate/severe impaired group). Thus, using the severity of kidney function categories as characteristics of the study subjects may not represent the complete results.

Dietary patterns were identified by using principal component analysis (PCA). Three dietary patterns were identified after an orthogonal varimax rotation based on eigenvalue > 1.4 and with the cut-off value of factor loading ≥0.30. Factor loadings are equivalent to a simple correlation between the food items and the extracted factor or pattern. Higher factor loadings indicate that the food shares more variance with that pattern. Factor scores were calculated for each pattern by summing up the intake of food items or groups weighed by their factor loadings, and thus each subject received a score for each identified dietary pattern [23]. For further analysis, dietary pattern scores were divided into tertiles. A multivariable linear regression analysis was performed to examine the independent variables associated with testosterone levels and the relationship across tertiles of dietary pattern scores with testosterone levels and kidney function biomarkers. The data were presented as regression coefficient (β) and 95% confidence interval (95% CI). Meanwhile, a multivariable logistic regression analysis was used to identify the association of dietary pattern scores across tertiles with impaired kidney function and proteinuria severity. The data were presented as odds ratio (OR) with 95% CI. Three adjustment models were used: model 1 adjusted for age, model 2 adjusted for age and BMI, and model 3 adjusted for model 2 and further adjusted for education level, marital status, family income, smoking, drinking alcohol, sleeping, physical activity, cardiovascular disease, hypertension, diabetes, and CRP levels. A *P*-value < 0.05 was considered statistically significant.

## Results

### Characteristics of the subjects

Among 21,376 men aged ≥40 years old (mean 51.9 ± 10.0 years), men with at least mild impaired kidney function (*n* = 19,519) had a mean of eGFR 77.7 ± 8.6 mL/min/1.73 m^2^ (data not shown). Men in the lowest tertile (T1) of eGFR tended to be older, heavier, and had higher prevalence of cardiovascular disease, hypertension, and diabetes mellitus (57.5, 46.7, and 44.6% respectively, *P* <  0.001) than those in the higher tertiles (T2 and T3) of eGFR (Table 1). In contrast, men in the lowest tertile of eGFR had lower serum testosterone levels compared to those in the higher tertiles of eGFR (4.6 ± 1.3 vs. 4.8 ± 1.4 and 5.4 ± 1.9 nmol/L in T1 vs. T2 and T3, *P* = 0.001).

**Table 1:** Characteristics of the subjects across tertiles of eGFR, *n* = 21,376 ^a^

	Total	Tertile of eGFR ^b^			*P* ^c^
		T1 (*n* = 7,128)	T2 (*n* = 7,149)	T3 (*n* = 7,099)	
Demographic data, lifestyle, and medical history					
Age, years	51.9 ± 10.0	58.0 ± 10.9	50.7 ± 8.5	47.1 ± 6.7	< 0.001
Education level, *n* (%)					< 0.001
< High school	3583 (17.0)	1898 (53.0)	1012 (28.2)	673 (18.8)	
High school	9354 (44.4)	2854 (30.5)	3169 (33.9)	3331 (35.6)	
> High school	8150 (38.6)	2254 (27.7)	2885 (35.4)	3011 (36.9)	
Marital status, *n* (%)					< 0.001
Never married	767 (3.8)	148 (19.3)	247 (32.2)	372 (48.5)	
Married	18412 (91.0)	6134 (33.3)	6207 (33.7)	6071 (33.0)	
Widowed/divorced	1048 (5.2)	455 (43.4)	320 (30.5)	273 (26.1)	
Family income, *n* (%)					< 0.001
< 800,000 NTD	7395 (37.7)	2975 (40.2)	2295 (31.0)	2125 (28.8)	
810,000–1.6 million NTD	8197 (41.8)	2338 (28.5)	2873 (35.1)	2986 (36.4)	
> 1.61 million NTD	4036 (20.6)	1103 (27.3)	1471 (36.5)	1462 (36.2)	
Smoking, *n* (%)					< 0.001
Never	11469 (55.4)	4017 (35.0)	3816 (33.3)	3636 (31.7)	
Past	3140 (15.2)	1120 (35.7)	1078 (34.3)	942 (30.0)	
Current	6086 (29.4)	1728 (28.4)	2046 (33.6)	2312 (38.0)	
Drinking alcohol, *n* (%)					0.001
No	14648 (73.0)	4950 (33.8)	4856 (33.1)	4842 (33.1)	
Yes	5405 (27.0)	1678 (31.1)	1855 (34.3)	1872 (34.6)	
Sleeping, *n* (%)					< 0.001
< 6 h	4828 (23.1)	1747 (36.2)	1572 (32.6)	1509 (31.2)	
6–7 h	10776 (51.5)	3407 (31.6)	3681 (34.2)	3688 (34.2)	
> 7 h	5324 (25.4)	1798 (33.8)	1753 (32.9)	1774 (33.3)	
Physical activity, *n* (%)					0.07
No	12554 (66.9)	4066 (32.4)	4213 (33.6)	4275 (34.0)	
Yes	6199 (33.1)	2073 (33.4)	2118 (34.2)	2008 (32.4)	
Cardiovascular disease, *n* (%)	1298 (6.5)	746 (57.5)	329 (25.3)	223 (17.2)	< 0.001
Hypertension, *n* (%)	6533 (44.0)	3051 (46.7)	1941 (29.7)	1541 (23.6)	< 0.001
Diabetes mellitus, *n* (%)	2356 (11.0)	1052 (44.6)	666 (28.3)	638 (27.1)	< 0.001
Anthropometric measurements					
BMI (kg/m^2^)	24.7 ± 3.1	24.9 ± 3.0	24.6 ± 3.0	24.5 ± 3.2	< 0.001
Systolic pressure (mmHg)	124.7 ± 16.7	128.1 ± 17.9	124.0 ± 16.2	122.0 ± 15.4	< 0.001
Diastolic pressure (mmHg)	76.9 ± 11.2	77.9 ± 11.5	76.8 ± 11.1	75.9 ± 11.0	< 0.001
Biochemical data					
FBG (mmol/L)	6.0 ± 1.3	6.1 ± 1.5	6.0 ± 1.2	5.9 ± 1.3	< 0.001
TG (mmol/L)	1.7 ± 1.2	1.7 ± 1.1	1.6 ± 1.1	1.7 ± 1.4	0.33
TC (mmol/L)	5.3 ± 0.9	5.3 ± 0.9	5.3 ± 0.9	5.2 ± 0.9	0.001
HDL-C (mmol/L)	1.4 ± 0.3	1.3 ± 0.3	1.4 ± 0.3	1.4 ± 0.3	0.001
LDL-C (mmol/L)	3.2 ± 0.8	3.2 ± 0.8	3.2 ± 0.8	3.1 ± 0.8	< 0.001
TC/HDL-C ratio	4.0 ± 0.9	4.0 ± 0.9	4.0 ± 0.9	3.9 ± 0.9	< 0.001
Testosterone (nmol/L)^d^	5.0 ± 1.6	4.6 ± 1.3	4.8 ± 1.4	5.4 ± 1.9	0.001
CRP (nmol/L)	23.2 ± 47.5	25.7 ± 48.9	22.3 ± 54.3	21.3 ± 37.2	< 0.001
Kidney function biomarkers					
BUN (mmol/L)	5.4 ± 1.4	6.0 ± 1.8	5.2 ± 1.1	4.9 ± 1.0	< 0.001
Creatinine (μmol/L)	100.8 ± 19.6	113.5 ± 29.0	98.4 ± 5.3	90.4 ± 4.1	< 0.001
eGFR (mL/min/1.73 m^2^)	75.4 ± 11.3	63.0 ± 7.7	74.5 ± 3.0	83.2 ± 3.4	< 0.001
Urinary protein, *n* (%)					< 0.001
+ 1	20260 (94.8)	6481 (32.0)	6909 (34.1)	6870 (33.9)	
+ 2	641 (3.0)	311 (48.5)	163 (25.4)	167 (26.1)	
+ 3	303 (1.4)	188 (62.0)	63 (20.8)	52 (17.2)	
+ 4	172 (0.8)	148 (86.1)	14 (8.1)	10 (5.8)	

*NTD* new taiwan dollar, *BMI* body mass index, *FBG* fasting blood glucose, *TG* triglycerides, *TC* total cholesterol, *HDL-C* high density lipoprotein-cholesterol, *LDL-C* low density lipoprotein-cholesterol, *TC/HDL-C* total cholesterol-to-high density lipoprotein-cholesterol, *CRP* C-reactive protein, *BUN* blood urea nitrogen, *eGFR* estimated glomerular filtration rate

^a^ Continuous data are presented as mean ± SD, and categorical data are presented as number (percentage)

^b^ Tertiles of eGFR, T1: ≤ 71.7 mL/min/1.73 m^2^; T2: 71.8–81.2 mL/min/1.73 m^2^; T3: ≥ 81.3 mL/min/1.73 m^2^

^c^ *P* was analyzed using general linear model for continuous variables and chi-square test for categorical variables

^d^ Testosterone: all, *n* = 256; T1, *n* = 78; T2, *n* = 91; T3, *n* = 87

### Dietary patterns

Table 2 shows three dietary patterns identified by PCA. The cumulative percentage of variance explained by three factors was 34.1% (13.8, 11.6, and 8.7% for each factor, respectively). The three dietary patterns were named based on the interpretation and components of the pattern structure. The first factor was named as the fried-processed dietary pattern and characterized by frequent consumption of ten food groups: deep-fried foods, preserved or processed foods, sauce, organ meats, sugar-sweetened beverages, meats, jam or honey, fried rice or flour products, instant noodles, and eggs. The second factor was named as the vege-seafood dietary pattern and consisted of six food groups: dark-colored vegetables, light-colored vegetables, oil added vegetables or salad, seafood, legumes or beans, and rice or flour products. The last factor was named as the dairy-grain dietary pattern and consisted of six food groups: dairy products, milk, bread, root crops, fruits, and whole grains. Fruits, meats, and root crops had a factor-loading ≥0.30 in the fried-processed and vege-seafood dietary patterns; while jam or honey was appeared in the vege-seafood and dairy-grain dietary patterns. However, these food groups could only belong to one factor (the one with a greater factor loading value).

**Table 2:** Factor loadings of three dietary patterns identified by principal component analysis ^a^

	Fried-processed dietary pattern	Vege-seafood dietary pattern	Dairy-grain dietary pattern
Dark-colored vegetables	–	0.756	–
Light-colored vegetables	–	0.742	–
Fruits	–	–	0.403
Root crops	–	–	0.47
Legumes/beans	–	0.431	–
Fried rice/flour products	0.47	–	–
Preserved/processed foods	0.599	–	–
Organ meats	0.501	–	–
Sauce	0.567	–	–
Instant noodles	0.424	–	–
Deep-fried foods	0.663	–	–
Meats	0.485	–	–
Eggs	0.39	–	–
Seafood	–	0.444	–
Dairy products	–	–	0.657
Milk	–	–	0.586
Bread	–	–	0.499
Whole grains	–	–	0.392
Sugar-sweetened beverages	0.501	–	–
Jam or honey	0.478	–	–
Rice or flour products	–	0.398	–
Oil added vegetables/salad	–	0.567	–

^a^ Factor loading below 0.30 was not shown in the table for simplicity. The food group with smaller factor loading value was eliminated as appearing in more than one dietary factor

### Testosterone and kidney function

The association between serum testosterone levels and kidney function is demonstrated in Table 3. After multivariable adjustment, serum testosterone levels among men in the lower tertiles (T1 and T2) of eGFR were significantly decreased by approximately 0.8 and 0.9 nmol/L (β = − 0.80, 95% CI: − 1.40, − 0.20 and β = − 0.88, 95% CI: − 1.43, − 0.33, *P* <  0.05) compared to those in the highest tertile (T3) of eGFR. Furthermore, BMI was negatively correlated with testosterone levels (β = − 0.11, 95% CI: − 0.19, − 0.04, *P* <  0.01), particularly obese men had significantly decreased testosterone levels (β = − 0.88, 95% CI: − 1.52, − 0.25, *P* <  0.01) compared to men with normal BMI (data not shown). TG and TC/HDL-C ratio were also negatively associated with testosterone levels (β = − 0.51, 95% CI: − 0.77, − 0.24 and β = − 0.42, 95% CI: − 0.75, − 0.08, *P* <  0.05). Correspondingly, age-adjusted or age and BMI-adjusted linier regression showed that serum testosterone levels were positively associated with eGFR levels (β = 0.83, 95% CI 0.17, 1.49 and β = 0.68, 95% CI: 0.01, 1.36, *P* <  0.05) (Additional file 1: Table S1). Moreover, after multivariable adjustment, age and overweight or obese BMI were negatively correlated with eGFR (β = − 0.57, 95% CI: − 0.59, − 0.55, β = − 1.17, 95% CI: − 1.63, − 0.71, and β = − 1.18, 95% CI: − 1.55, − 0.80, *P* <  0.001 for all). All blood lipid components were negatively associated with eGFR levels (*P* <  0.001), but HDL-C levels were positively associated with eGFR levels (β = 1.82, 95% CI: 1.26, 2.39, *P* <  0.001) (Additional file 1: Table S1).

**Table 3:** Multivariable linear regression analysis of independent variables affecting serum testosterone levels (nmol/L), *n* = 256

	Model 1 ^a^		Model 2 ^b^		Model 3 ^c^	
	β (95% CI)	*P*	β (95% CI)	*P*	β (95% CI)	*P*
Age (years)	0.01 (−0.02, 0.03)	0.63	0.00 (− 0.02, 0.03)	0.78	0.01 (− 0.03, 0.04)	0.78
BMI (kg/m^2^)	− 0.11 (− 0.18, − 0.05)	< 0.001	− 0.11 (− 0.18, − 0.05)	< 0.001	− 0.11 (− 0.19, − 0.04)	0.004
FBG (mmol/L)	−0.15 (− 0.32, 0.01)	0.07	−0.09 (− 0.26, 0.08)	0.30	−0.21 (− 0.53, 0.11)	0.20
TG (mmol/L)	−0.49 (− 0.68, − 0.30)	< 0.001	−0.42 (− 0.61, − 0.22)	< 0.001	−0.51 (− 0.77, − 0.24)	< 0.001
TC (mmol/L)	−0.12 (− 0.34, 0.09)	0.25	−0.07 (− 0.28, 0.14)	0.50	−0.14 (− 0.42, 0.13)	0.31
HDL-C (mmol/L)	0.69 (0.00, 1.37)	0.05	0.36 (−0.34, 1.06)	0.31	0.53 (−0.29, 1.35)	0.20
LDL-C (mmol/L)	0.04 (−0.21, 0.28)	0.77	0.09 (−0.15, 0.32)	0.48	−0.02 (− 0.34, 0.31)	0.93
TC/HDL-C ratio	−0.36 (− 0.59, − 0.13)	0.002	−0.23 (− 0.48, 0.01)	0.06	−0.42 (− 0.75, − 0.08)	0.014
BUN (mmol/L)	−0.08 (− 0.25, 0.10)	0.38	−0.07 (− 0.24, 0.10)	0.40	0.01(− 0.19, 0.21)	0.92
Creatinine (μmol/L)	−0.03 (− 0.04, − 0.01)	0.015	−0.02 (− 0.04, 0.00)	0.049	−0.02 (− 0.04, 0.01)	0.13
eGFR (mL/min/1.73 m^2^)						
T3	Ref		Ref		Ref	
T2	−0.72(− 1.18, − 0.26)	0.002	− 0.62 (− 1.08, − 0.17)	0.007	−0.88 (− 1.43, − 0.33)	0.002
T1	− 0.94 (− 1.44, − 0.44)	0.001	−0.81 (− 1.31, − 0.31)	0.002	−0.80 (− 1.40, − 0.20)	0.010

*BMI* body mass index, *FBG* fasting blood glucose, *TG* triglycerides, *TC* total cholesterol, *HDL-C* high density lipoprotein-cholesterol, *LDL-C* low density lipoprotein-cholesterol, *TC/HDL-C* total cholesterol-to-high density lipoprotein-cholesterol, *BUN* blood urea nitrogen, *eGFR* estimated glomerular filtration rate

^a^ Model 1: adjusted for age

^b^ Model 2: adjusted for age and BMI

^c^ Model 3: adjusted for model 2 and for education level, marital status, family income, smoking, drinking alcohol, sleeping, physical activity, cardiovascular disease, hypertension, diabetes, and CRP levels

### Dietary patterns, testosterone levels, and severity of impaired kidney function

Table 4 shows the association between dietary pattern scores across tertiles and serum testosterone levels. After multivariable adjustment, the fried-processed dietary pattern was significantly associated with decreased testosterone levels by 0.8 nmol/L (β = − 0.78, 95% CI: − 1.40, − 0.16, *P* <  0.05) and reduced testosterone-to-triglycerides (T/TG) ratio by 1.8 units (β = − 1.76, 95% CI: − 2.99, − 0.53, *P* <  0.01) among men in the higher tertile (T2) of dietary pattern scores compared to those in the lowest tertile (T1) of dietary pattern scores. However, the vege-seafood or dairy-grain dietary pattern was not significantly correlated with testosterone levels or T/TG ratio. Moreover, the fried-processed dietary pattern was positively associated with BUN and blood creatinine levels (β = 0.09, 95% CI: 0.04, 0.15 and β = 0.94, 95% CI: 0.08, 1.80, *P* <  0.05), but negatively associated with eGFR (β = − 0.64, 95% CI: − 1.05, − 0.24, *P* <  0.01) among men in the higher tertile (T2) of dietary pattern scores. In contrast, compared with the lowest tertile (T1), the higher tertiles (T2 and T3) of vege-seafood dietary pattern scores were negatively correlated with creatinine levels, but positively correlated with eGFR levels.

**Table 4:** Multivariable linear regression analysis of across tertiles of dietary pattern scores associated with serum testosterone and kidney function biomarkers ^a^

	Fried-processed dietary pattern				Vege-seafood dietary pattern			
	T2	*P*	T3	*P*	T2	*P*	T3	*P*
	β (95% CI)		β (95% CI)		β (95% CI)		β (95% CI)	
Testosterone (nmol/L)	−0.78 (− 1.40, − 0.16)	0.014	−0.19 (− 0.86, 0.48)	0.58	−0.05 (− 0.82, 0.71)	0.89	0.04 (− 0.73, 0.81)	0.92
T/TG ratio	−1.76 (−2.99, − 0.53)	0.005	−0.81 (− 2.14, 0.53)	0.24	0.54 (− 0.99, 2.07)	0.48	0.94 (− 0.60, 2.49)	0.23
BUN (mmol/L)	0.09 (0.04, 0.15)	0.001	0.06 (−0.00, 0.12)	0.06	−0.00 (− 0.06, 0.05)	0.89	− 0.03 (− 0.09, 0.03)	0.36
Creatinine (μmol/L)	0.94 (0.08, 1.80)	0.031	0.21 (−0.67, 1.09)	0.64	−1.03 (− 1.89, − 0.17)	0.018	−1.47 (−2.32, − 0.61)	0.001
eGFR (mL/min/1.73 m^2^)	−0.64 (− 1.05, − 0.24)	0.002	−0.39 (− 0.81, 0.03)	0.07	0.46 (0.05, 0.86)	0.027	1.01 (0.60, 1.41)	< 0.001

*T/TG* testosterone-to-triglycerides, *BUN* blood urea nitrogen, *eGFR* estimated glomerular filtration rate

^a^ Data were analyzed by model 3 adjusted for age, BMI, education level, marital status, family income, smoking, drinking alcohol, sleeping, physical activity, cardiovascular disease, hypertension, diabetes, and CRP levels using T1 as the reference group (*n* = 21,376, except for testosterone levels and T/TG ratio, *n* = 256). Dairy-grain dietary pattern is not listed in the table because there was no association between the dairy-grain dietary pattern and all the variables above

**Fig. 2:**
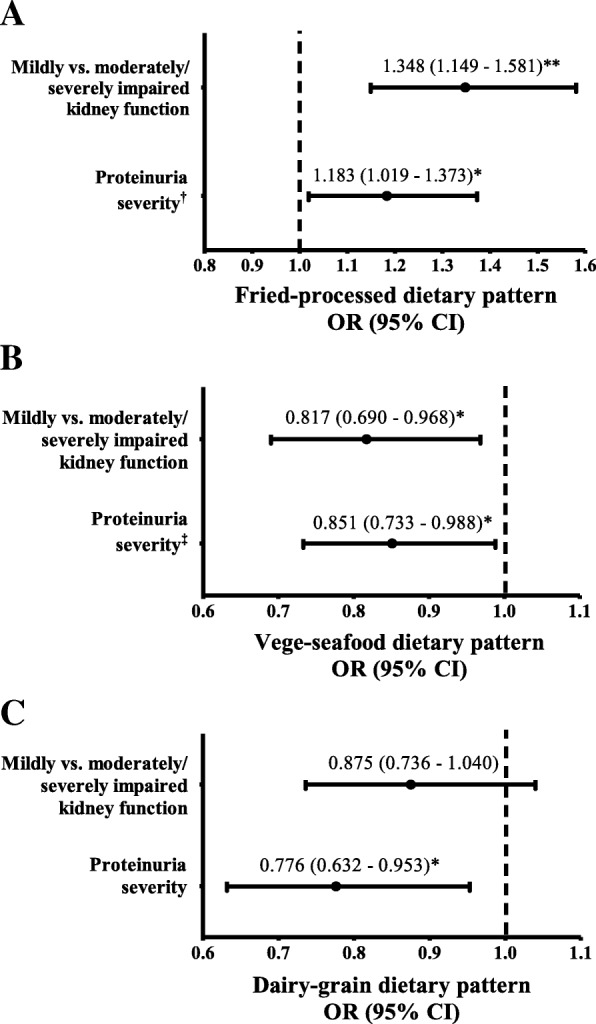
Multivariable logistic regression analysis for predicting impaired kidney function and proteinuria severity associated with (**a**) fried-processed dietary pattern, (**b**) vege-seafood dietary pattern, and (**c**) dairy-grain dietary pattern in tertile 3 of dietary pattern scores compared to tertile 1 as the reference group (*n* = 21,376). Data are expressed as odd ratio (OR) and 95% confidence interval (95% CI) in the parentheses and adjusted for age, BMI, education level, marital status, family income, smoking, drinking alcohol, sleeping, physical activity, cardiovascular disease, hypertension, diabetes, and CRP levels (model 3) except ^†^adjusted for age (model 1) and ^‡^adjusted for age and BMI (model 2). Proteinuria severity analyzed as dummy variables and used one plus (+ 1) as a reference. *P < 0.05, **P < 0.01

Furthermore, a multivariable logistic regression analysis revealed that men in the highest tertile (T3) of fried-processed dietary pattern scores were significantly associated with 35 and 18% increased risks of moderate/severe impaired kidney function and proteinuria severity respectively, compared to those in the lowest tertile (T1) (Fig. 2). In contrast, the highest tertile (T3) of vege-seafood dietary pattern scores had inverse association with moderate/severe impaired kidney function (OR = 0.82, 95% CI: 0.69, 0.97, *P* <  0.05) and proteinuria severity (OR = 0.85, 95% CI: 0.73, 0.99, *P* <  0.05). However, after adjusting for model 3, the vege-seafood dietary pattern was not significantly correlated with proteinuria severity (OR = 0.93, 95% CI: 0.77, 1.12, *P* = 0.46, data not shown). Similarly, the association between proteinuria severity and the fried-processed dietary pattern was altered after further adjustment in model 2 (OR = 1.06, 95% CI: 0.91, 1.23, *P* = 0.46, data not shown). Moreover, the highest tertile (T3) of dairy-grain dietary pattern scores were also significantly associated with reduced risk of proteinuria severity (OR = 0.78, 95% CI: 0.63, 0.95, *P* <  0.05), but not significantly correlated with the severity of impaired kidney function.

## Discussion

### Testosterone and kidney function

Our study found that men aged ≥40 years with lower tertiles of eGFR were associated with a decrease in serum testosterone levels (T2 and T1 β = − 0.80, 95% CI-1.40, − 0.20 and β = − 0.88, 95% CI -1.43, − 0.33, *P* <  0.05, respectively). The association between kidney function and testosterone levels in men has been reported in previous studies [7, 24–27]. Japanese adult men with low salivary testosterone levels (22.0 pg/mL, the 5th percentile) were associated with reduced eGFR (− 3.43 mL/min/1.73 m^2^, 95% CI: − 6.02, − 0.84 mL/min/1.73 m^2^) compared to those in the 90th percentile of salivary testosterone levels (156.9 pg/mL) [7]. In agreement, our study also found that 1 nmol/L increment of serum testosterone levels were correlated with increased eGFR levels by 0.7 mL/min/1.73 m^2^ after adjustment by age and BMI. However, there are other confounders that potentially influenced these results (Additional file 1: Table S1). Furthermore, our study did not find any association between serum testosterone levels and the risk of impaired kidney function or proteinuria severity (Additional file 1: Table S2) likely due to the small number of men who had serum testosterone data.

The possible mechanisms for the association between impaired kidney function and low testosterone levels are multi-factorial including uremia [28] and increased prolactin levels [29]. Comorbid conditions such as hypertension and diabetes may also contribute to reduce the levels of testosterone [30]. Uremia, commonly seen in patients with impaired kidney function, could also impair the hypothalamic-pituitary-gonadal axis and reduce testosterone production [31]. In addition, a previous study showed that prolactin levels were inversely associated with kidney function (eGFR) due to the reduction of prolactin clearance by the kidney [29]. Both uremia and prolactin negatively affect gonadal function by inhibiting the secretion of gonadotropin-releasing hormone and luteinizing hormone for testosterone production in the Leydig cells [32]. Our study also found that TG and TC/HDL-C ratio were independently associated with decreased testosterone levels. Consistent with our findings, previous studies have shown a negative correlation between testosterone levels and TG levels in men with impaired kidney function [25, 33]. Decreased testosterone levels by 41%, accompanied by 58% increase in TG levels were reported in men with advanced CKD stage compared to those in early CKD stage [25]. Additionally, lower testosterone levels and higher TG levels contribute to a rapid loss of kidney function [34] and all-cause mortality [5, 6, 24, 27]. Therefore, maintaining normal testosterone levels might have a beneficial effect to prevent a rapid decline in kidney function and CKD related outcomes.

### Dietary patterns and testosterone levels

To the best of our knowledge, our study is the first study to investigate the association between dietary patterns and testosterone levels in middle-aged and elderly men with impaired kidney function. In this study, we demonstrated that the fried-processed dietary pattern was independently associated with decreased testosterone levels (β = − 0.78, 95% CI: − 1.40, − 0.16, *p* <  0.05) and T/TG ratio (β = − 1.76, 95% CI: − 2.99, − 0.53, *P* <  0.01). In our study, the fried-processed dietary pattern consisted of food groups that are high in calories, saturated fat, artificial sweets, and sodium. Previous prospective studies have described the association between low caloric diets and testosterone levels [35–37]. Plasma total testosterone levels have been reported to be significantly increased after an 8-week low caloric diet (850–900 kcal/d) in obese nondiabetic men [35]. In a clinical trial of overweight or obese men on low fat and energy-restricted diet (~ 1600 kcal/d) for 52 weeks, significant improvements in serum testosterone levels were found [13]. Limited published articles have investigated specific dietary patterns with male testosterone levels. A recent cross-sectional study in 125 Taiwanese men reported that higher adherence to a typical high-calorie Western diet was associated with decreased testosterone level by 0.87 ng/mL [38]. However, another study found that men consuming vegetarian diets had no association with serum testosterone levels [14, 15]. Consistently, we did not find an association between vege-seafood dietary pattern and testosterone levels. Elevated testosterone levels seem to occur in overweight or obese men following a low-fat or low-calorie diet, presumably due to weight loss and less aromatization of testosterone to estradiol [39]. However, the potential links between dietary patterns and testosterone levels are unclear, but might be due to Western diet-related insulin resistance [38]. Insulin stimulates testosterone production and insulin resistance may impair this process in the Leydig cells [40]. Whereas, previous studies were conducted in smaller samples, hence further larger prospective studies are needed to assess the effect of dietary patterns on male testosterone levels.

### Dietary patterns and kidney function

Our study also found that the fried-processed dietary pattern was positively associated with increased risk of moderate/severe impaired kidney function and proteinuria severity. However, the association between the fried-processed dietary pattern with proteinuria severity was altered after further adjustment in model 2 (OR = 1.06, 95% CI: 0.91, 1.23, *P* = 0.46) and model 3 (OR = 1.02, 95% CI: 0.83, 1.25, *P* = 0.88). Previous studies have reported that subjects with higher adherence to typical Western dietary patterns were associated with increased creatinine levels and decreased eGFR levels [9–11, 41, 42]. The Nurses’ Health Study has found that high adherence to a Western dietary pattern, characterized by high intake of meat, processed meat, saturated fat, and sweets, was positively correlated with rapid eGFR decline and albuminuria [9–11]. Two recent studies among Singaporean Chinese and US adults also addressed that red meat and processed meat were associated with the onset of CKD and kidney failure [41, 42]. In addition, a Southern dietary pattern, consisted of primarily fried-processed foods, organ meats, and sweetened beverages, was correlated with increased mortality in CKD subjects [43]. In general, animal-based protein with high phosphorus and biological value could produce acid metabolites and increase dietary acid load, which may provoke the kidney to excrete excess acid. A dietary acid load was found to be positively associated with the progression of advanced CKD stage and albuminuria [44, 45]. In addition, our study also showed that there was a negative association between dairy-grain dietary pattern and proteinuria severity. Differences in the direction between protein-rich dietary patterns and kidney dysfunction could possibly be explained by the composition of amino acids and fatty acids between non-dairy and dairy foods. Dairy foods contain large amounts of branched-chain amino acids, which have a lesser effect on kidney function [46].

In our study, the vege-seafood dietary pattern had an inverse association with the severity of impaired kidney function and proteinuria. Consistent with our findings, a cross-sectional study in Taiwan demonstrated that a dietary pattern with high intake of fish and vegetables had a positive association with kidney function [12]. Diets rich in whole grains, fruits, and low-fat dairy products were also correlated with lower risks of albuminuria [46] and mortality [47]. Moreover, a large prospective cohort study reported that plant sources of protein; like legumes/nuts, dairy protein, fish, and seafood reduced the risk of CKD [42]. Plant-based protein is predominantly alkaline-forming. Diets high in plant-based protein, fruits, and vegetables have been reported to be associated with higher blood bicarbonate levels, lower blood pressure, reduced renal acid load, decreased uremic toxin and inflammatory markers, and reduced serum creatinine and proteinuria [46, 48–51]. Seafood that is rich in polyunsaturated fatty acids has anti-inflammatory properties, which may also improve kidney function [52].

### Strengths and limitations

The strength of this study is a relatively large study representative that was used to determine the association between dietary patterns and severity of impaired kidney function in men. Additionally, we used the CKD-EPI equation to estimate eGFR levels due to its higher accuracy than other equations [19]. However, present study had certain limitations. First, due to the cross-sectional study design, our study cannot establish a causal relationship between dietary patterns and severity of impaired kidney function. A temporal relationship may occur as we observed in the present study, and could be affected by the dietary advice given to all participants due to their disease conditions. Furthermore, subjects who had received health screenings may have been health-conscious and changed their dietary habits. Second, data availability of other male sex hormones such as sex hormone binding globulin, free and albumin-bound testosterone are limited. Measurement of other testosterone forms may be more sensitive to determine male sex hormone status. Moreover, we used the data from the full sample to derive dietary patterns, whereas the testosterone analysis was conducted on a very small subset (1.2% of the full sample). Third, the self-reported food frequency questionnaire may have self-reporting bias and measurement errors. Although a food frequency questionnaire is the most common method used to evaluate dietary patterns, given its feasibility and affordability in nutritional epidemiological studies, we cannot neglect that self-reported dietary assessment methods are known for both omission (e.g. unreported or underreporting food that was eaten) and intrusion errors (e.g. lack of memories when reporting). However, in the present study, we used a validated and standardized semi-quantitative food frequency questionnaire that has been published elsewhere [21, 22]. Nevertheless, future studies using rigorous scientific methods such as randomized trials are needed to investigate the role of dietary patterns in the development of chronic diseases. Fourth, due to the lack of quantitative data concerning urine protein, we used urinary protein dipstick as a marker of proteinuria instead of measuring albuminuria, which is more standardized measurement of kidney damage. Moreover, clinically diagnosed CKD was defined as a patient with eGFR < 60 ml/min/1.73 m^2^ for ≥3 months, with or without persistent albuminuria [20]. Although MJ Health Institute provides periodic health screenings to its members, not all subjects had an annual examination. Hence, we cannot identify clinically diagnosed CKD based on one single measurement only. Therefore, our results may not necessarily reflect the clinically diagnosed CKD subjects, and not generalize to those with impaired kidney function and without clinical proteinuria. Finally, some potential confounders such as total energy and protein intake might affect the study findings.

## Conclusion

In conclusion, our findings suggest that frequent consumption of fried-processed dietary pattern is associated with decreased testosterone levels and T/TG ratio as well as increased risk of impaired kidney function and proteinuria severity. In contrast, the vege-seafood dietary pattern is negatively associated with moderately/severely impaired kidney function and proteinuria severity, but has no association with either testosterone levels or T/TG ratio. Further studies with prospective measurements are needed to elucidate the association between dietary patterns and testosterone levels in men with impaired kidney function.

## Additional file

Additional file 1: **Table S1.** Multivariable linear regression analysis of independent variables affecting eGFR (mL/min/1.73 m^2^), *n* = 21,376. **Table S2.** Multivariable logistic regression analysis for predicting moderate/severe impaired kidney function and proteinuria severity associated with serum testosterone and lipid profiles ^**a**^. (DOCX 27 kb)

## Data Availability

The data that support the findings of this study are available from Mei Jau (MJ) Health Institute, but restricted for research use only. The data are not publicly available. Data are available from the authors upon reasonable request and with permission of MJ Health Institute.

## Abbreviations

BMI: Body mass index
BUN: Blood urea nitrogen
CKD: Chronic kidney disease
CRP: C-reactive protein
CVD: Cardiovascular disease
DBP: Diastolic blood pressure
eGFR: Estimated glomerular filtration rate
FBG: Fasting blood glucose
HDL-C: High density lipoprotein-cholesterol
LDL-C: Low density lipoprotein-cholesterol
PCA: Principal component analysis
SBP: Systolic blood pressure
TC: Total cholesterol
TC/HDL-C: Total cholesterol-to-HDL-C ratio
TG: Triglycerides

## References

[1] Wen CP, Cheng TY, Tsai MK, Chang YC, Chan HT, Tsai SP (2008). All-cause mortality attributable to chronic kidney disease: a prospective cohort study based on 462 293 adults in Taiwan. Lancet..

[2] Saran R, Robinson B, Abbott KC, Agodoa LY, Albertus P, Ayanian J (2017). US renal data system 2016 annual data report: epidemiology of kidney disease in the United States. Am J Kidney Dis.

[3] Yang WC, Hwang SJ, Taiwan Society of Nephrology (2008). Incidence, prevalence and mortality trends of dialysis end-stage renal disease in Taiwan from 1990 to 2001: the impact of national health insurance. Nephrol Dial Transplant.

[4] Schmidt A, Luger A, Horl WH (2002). Sexual hormone abnormalities in male patients with renal failure. Nephrol Dial Transplant.

[5] Yilmaz MI, Sonmez A, Qureshi AR, Saglam M, Stenvinkel P, Yaman H (2011). Endogenous testosterone, endothelial dysfunction, and cardiovascular events in men with nondialysis chronic kidney disease. Clin J Am Soc Nephrol.

[6] Carrero JJ, Qureshi AR, Parini P, Arver S, Lindholm B, Barany P (2009). Low serum testosterone increases mortality risk among male dialysis patients. J Am Soc Nephrol.

[7] Kurita N, Horie S, Yamazaki S, Otani K, Sekiguchi M, Onishi Y (2016). Low testosterone levels and reduced kidney function in Japanese adult men: the locomotive syndrome and health outcome in Aizu cohort study. J Am Med Dir Assoc.

[8] Schulze MB, Martinez-Gonzalez MA, Fung TT, Lichtenstein AH, Forouhi NG (2018). Food based dietary patterns and chronic disease prevention. BMJ..

[9] Knight EL, Stampfer MJ, Hankinson SE, Spiegelman D, Curhan GC (2003). The impact of protein intake on renal function decline in women with normal renal function or mild renal insufficiency. Ann Intern Med.

[10] Lin JL, Fung TT, Hu FB, Curhan GC (2011). Association of dietary patterns with albuminuria and kidney function decline in older white women: a subgroup analysis from the nurses’ health study. Am J Kidney Dis.

[11] Lin JL, Hu FB, Curhan GC (2010). Associations of diet with albuminuria and kidney function decline. Clin J Am Soc Nephrol.

[12] Hsu CC, Jhang HR, Chang WT, Lin CH, Shin SJ, Hwang SJ (2014). Associations between dietary patterns and kidney function indicators in type 2 diabetes. Clin Nutr.

[13] Moran LJ, Brinkworth GD, Martin S, Wycherley TP, Stuckey B, Lutze J (2016). Long-term effects of a randomised controlled trial comparing high protein or high carbohydrate weight loss diets on testosterone, SHBG, erectile and urinary function in overweight and obese men. PLoS One.

[14] Belanger A, Locong A, Noel C, Cusan L, Dupont A, Prevost J (1989). Influence of diet on plasma steroids and sex hormone-binding globulin levels in adult men. J Steroid Biochem.

[15] Key TJ, Roe L, Thorogood M, Moore JW, Clark GM, Wang DY (1990). Testosterone, sex hormone-binding globulin, calculated free testosterone, and oestradiol in male vegans and omnivores. Br J Nutr.

[16] Giles TD, Materson BJ, Cohn JN, Kostis JB (2009). Definition and classification of hypertension: an update. J Clin Hypertens (Greenwich).

[17] American Diabetes Association (2014). Diagnosis and classification of diabetes mellitus. Diabetes Care.

[18] Wu DM, Pai L, Chu NF, Sung PK, Lee MS, Tsai JT (2001). Prevalence and clustering of cardiovascular risk factors among healthy adults in a Chinese population: the MJ health screening center study in Taiwan. Int J Obes.

[19] Levey AS, Stevens LA, Schmid CH, Zhang YL, Castro AF, Feldman HI (2009). A new equation to estimate glomerular filtration rate. Ann Intern Med.

[20] Kidney Disease Improving Global Outcome (2013). Chapter 1: definition and classification of CKD. Kidney Int Suppl.

[21] Lyu LC, Lin CF, Chang FH, Chen HF, Lo CC, Ho HF (2007). Meal distribution, relative validity and reproducibility of a meal-based food frequency questionnaire in Taiwan. Asia Pac J Clin Nutr.

[22] Muga MA, Owili PO, Hsu CY, Rau HH, Chao JCJ (2016). Association between dietary patterns and cardiovascular risk factors among middle-aged and elderly adults in Taiwan: a population-based study from 2003 to 2012. PLoS One.

[23] Kleinbaum DG, Kupper LL, Muller KE (1988). Variable reduction and factor analysis. In: applied regression analysis and other multivariable methods.

[24] Khurana KK, Navaneethan SD, Arrigain S, Schold JD, Nally JV, Shoskes DA (2014). Serum testosterone levels and mortality in men with CKD stages 3-4. Am J Kidney Dis.

[25] Hylander B, Lehtihet M (2015). Testosterone and gonadotropins but not SHBG vary with CKD stages in young and middle aged men. Basic Clin Androl.

[26] Fugl-Meyer KS, Nilsson M, Hylander B, Lehtihet M (2017). Sexual function and testosterone level in men with conservatively treated chronic kidney disease. Am J Mens Health.

[27] Carrero JJ, Qureshi AR, Nakashima A, Arver S, Parini P, Lindholm B (2011). Prevalence and clinical implications of testosterone deficiency in men with end-stage renal disease. Nephrol Dial Transplant.

[28] Holley JL (2004). The hypothalamic-pituitary axis in men and women with chronic kidney disease. Adv Chronic Kidney Dis.

[29] Carrero JJ, Kyriazis J, Sonmez A, Tzanakis I, Qureshi AR, Stenvinkel P (2012). Prolactin levels, endothelial dysfunction, and the risk of cardiovascular events and mortality in patients with CKD. Clin J Am Soc Nephrol.

[30] Grossmann M, Gianatti EJ, Zajac JD (2010). Testosterone and type 2 diabetes. Curr Opin Endocrinol Diabetes Obes.

[31] Dousdampanis P, Trigka K, Fourtounas C, Bargman JM (2014). Role of testosterone in the pathogenesis, progression, prognosis and comorbidity of men with chronic kidney disease. Ther Apher Dial.

[32] Iglesias P, Carrero JJ, Diez JJ (2012). Gonadal dysfunction in men with chronic kidney disease: clinical features, prognostic implications and therapeutic options. J Nephrol.

[33] Gungor O, Kircelli F, Carrero JJ, Asci G, Toz H, Tatar E (2010). Endogenous testosterone and mortality in male hemodialysis patients: is it the result of aging?. Clin J Am Soc Nephrol.

[34] Wang YN, Qiu XL, Lv LS, Wang CX, Ye ZC, Li SM (2016). Correlation between serum lipid levels and measured glomerular filtration rate in Chinese patients with chronic kidney disease. PLoS One.

[35] Khoo J, Piantadosi C, Worthley S, Wittert GA (2010). Effects of a low-energy diet on sexual function and lower urinary tract symptoms in obese men. Int J Obes.

[36] Niskanen L, Laaksonen DE, Punnonen K, Mustajoki P, Kaukua J, Rissanen A (2004). Changes in sex hormone-binding globulin and testosterone during weight loss and weight maintenance in abdominally obese men with the metabolic syndrome. Diabetes Obes Metab.

[37] Schulte DM, Hahn M, Oberhauser F, Malchau G, Schubert M, Heppner C (2014). Caloric restriction increases serum testosterone concentrations in obese male subjects by two distinct mechanisms. Horm Metab Res.

[38] Hu TY, Chen YC, Lin P, Shih CK, Bai CH, Yuan KC (2018). Testosterone-associated dietary pattern predicts low testosterone levels and hypogonadism. Nutrients.

[39] Grossmann M (2018). Hypogonadism and male obesity: focus on unresolved questions. Clin Endocrinol.

[40] Pitteloud N, Hardin M, Dwyer AA, Valassi E, Yialamas M, Elahi D (2005). Increasing insulin resistance is associated with a decrease in Leydig cell testosterone secretion in men. J Clin Endocrinol Metab.

[41] Lew QLJ, Jafar TH, Koh HWL, Jin AZ, Chow KY, Yuan JM (2017). Red meat intake and risk of ESRD. J Am Soc Nephrol.

[42] Haring B, Selvin E, Liang M, Coresh J, Grams ME, Petruski-Ivleva N (2017). Dietary protein sources and risk for incident chronic kidney disease: results from the atherosclerosis risk in communities (ARIC) study. J Ren Nutr.

[43] Gutierrez OM, Muntner P, Rizk DV, McClellan WM, Warnock DG, Newby PK (2014). Dietary patterns and risk of death and progression to ESRD in individuals with CKD: a cohort study. Am J Kidney Dis.

[44] Banerjee T, Crews DC, Wesson DE, Tilea AM, Saran R, Rios-Burrows N (2015). High dietary acid load predicts ESRD among adults with CKD. J Am Soc Nephrol.

[45] Banerjee T, Crews DC, Wesson DE, Tilea A, Saran R, Rios-Burrows N (2014). Dietary acid load and chronic kidney disease among adults in the United States. BMC Nephrol.

[46] Nettleton JA, Steffen LM, Palmas W, Burke GL, Jacobs DR (2008). Associations between microalbuminuria and animal foods, plant foods, and dietary patterns in the multiethnic study of atherosclerosis. Am J Clin Nutr.

[47] Kelly JT, Palmer SC, Wai SN, Ruospo M, Carrero JJ, Campbell KL (2017). Healthy dietary patterns and risk of mortality and ESRD in CKD: a meta-analysis of cohort studies. Clin J Am Soc Nephrol.

[48] Goraya N, Simoni J, Jo CH, Wesson DE (2013). A comparison of treating metabolic acidosis in CKD stage 4 hypertensive kidney disease with fruits and vegetables or sodium bicarbonate. Clin J Am Soc Nephrol.

[49] Scialla JJ, Appel LJ, Wolf M, Yang W, Zhang XM, Sozio SM (2012). Plant protein intake is associated with fibroblast growth factor 23 and serum bicarbonate levels in patients with chronic kidney disease: the chronic renal insufficiency cohort study. J Renal Nutr.

[50] Chen X, Wei G, Jalili T, Metos J, Giri A, Cho ME (2016). The associations of plant protein intake with all-cause mortality in CKD. Am J Kidney Dis.

[51] Zhou J, Yuan WJ (2016). Effects of soy protein containing isoflavones in patients with chronic kidney disease: a systematic review and meta-analysis. Clin Nutr.

[52] Chrysohoou C, Pitsavos C, Panagiotakos D, Skoumas J, Lazaros G, Oikonomou E (2013). Long-term fish intake preserves kidney function in elderly individuals: the Ikaria study. J Renal Nutr..

