# Supplementary material for: Dietary patterns in relation to testosterone levels and severity of impaired kidney function among middle-aged and elderly men in Taiwan: a cross-sectional study
Source: Nutr J. 2019 Jul 27;18:42. doi: 10.1186/s12937-019-0467-x (PMC6660671; doi:10.1186/s12937-019-0467-x)
Supplement: Supplementary file 1 — Table S1. Multivariable linear regression analysis of independent variables affecting eGFR (mL/min/1.73 m2), n = 21,376. Table S2. Multivariable logistic regression analysis for predicting moderate/severe impaired kidney function and proteinuria severity associated with serum testosterone and lipid profiles a. (DOCX 27 kb) [file 12937_2019_467_MOESM1_ESM.docx]

**Dietary patterns in relation to testosterone levels and severity of impaired kidney function among middle-aged and elderly men in Taiwan: a cross-sectional study**

Adi Lukas Kurniawan ^1^, Chien-Yeh Hsu ^2,3^, Hsiao-Hsien Rau ^4^, Li-Yin Lin^1^, and Jane C-J Chao ^1,3,5*^

**Table S1.** Multivariable linear regression analysis of independent variables affecting eGFR (mL/min/1.73 m^2^), *n* = 21,376

| Independent variable | Model 1^a^ | | Model 2^b^ | | Model 3^c^ | |
| --- | --- | --- | --- | --- | --- | --- |
|  | β (95% CI) | *P* | β (95% CI) | *P* | β (95% CI) | *P* |
| Age (years) | -0.60 (-0.61, -0.59) | < 0.001 | -0.60 (-0.62, -0.59) | < 0.001 | -0.57 (-0.59, -0.55) | < 0.001 |
| BMI (kg/m^2^) | -0.29 (-0.33, -0.25) | < 0.001 | -0.29 (-0.33, -0.25) | < 0.001 | -0.20 (-0.25, -0.14) | < 0.001 |
| Normal ^d^ | Ref |  | Ref |  | Ref |  |
| Overweight ^e^ | -1.47 (-1.76, -1.18) | < 0.001 | -1.47 (-1.76, -1.18) | < 0.001 | -1.17 (-1.63, -0.71) | < 0.001 |
| Obese ^f^ | -1.96 (-2.30, -1.61) | < 0.001 | -1.96 (-2.30, -1.61) | < 0.001 | -1.18 (-1.55, -0.80) | < 0.001 |
| FBG (mmol/L) | -0.03 (-0.13, 0.07) | 0.51 | 0.08 (-0.02, 0.18) | 0.11 | 0.16 (-0.02, 0.33) | 0.08 |
| TG (mmol/L) | -0.47 (-0.57, -0.36) | < 0.001 | -0.31 (-0.42, -0.20) | < 0.001 | -0.33 (-0.47, -0.19) | < 0.001 |
| TC (mmol/L) | -0.64 (-0.79, -0.50) | < 0.001 | -0.59 (-0.73, -0.45) | < 0.001 | -0.70 (-0.89, -0.52) | < 0.001 |
| HDL-C (mmol/L) | 2.44 (2.02, 2.85) | < 0.001 | 1.72 (1.28, 2.15) | < 0.001 | 1.82 (1.26, 2.39) | < 0.001 |
| LDL-C (mmol/L) | -0.71 (-0.88, -0.55) | < 0.001 | -0.67 (-0.83, -0.51) | < 0.001 | -0.81 (-1.02, -0.60) | < 0.001 |
| TC/HDL-C ratio | -1.24 (-1.38, -1.10) | < 0.001 | -1.05 (-1.19, -0.90) | < 0.001 | -1.17 (-1.36, -0.98) | < 0.001 |
| Testosterone (nmol/L)^g^ | 0.83 (0.17, 1.49) | 0.014 | 0.68 (0.01, 1.36) | 0.046 | 0.62 (-0.18, 1.41) | 0.13 |
| eGFR: estimated glomerular filtration rate, BMI: body mass index, FBG: fasting blood glucose, TG: triglycerides, TC: total cholesterol, HDL-C: high density lipoprotein-cholesterol, LDL-C: low density lipoprotein-cholesterol, TC/HDL-C: total cholesterol-to-high density lipoprotein-cholesterol.  ^a^ Model 1: adjusted for age.  ^b^ Model 2: adjusted for age and BMI.  ^c^ Model 3: adjusted for model 2 and for education level, marital status, family income, smoking, drinking alcohol, sleeping, physical activity, cardiovascular disease, hypertension, diabetes, and CRP levels.  ^d^ Normal BMI: BMI < 24 kg/m^2^.  ^e^ Overweight BMI: 24 kg/m^2^ ≤ BMI < 27 kg/m^2^.  ^f^ Obese BMI: BMI ≥ 27 kg/m^2^.  ^g^ *n* = 256. | | | | | | |

**Table S2.** Multivariable logistic regression analysis for predicting moderate/severe impaired kidney function and proteinuria severity associated with serum testosterone and lipid profiles **^a^**

| Independent variable | Mild vs. moderate/severe impaired kidney function | | Proteinuria severity | |
| --- | --- | --- | --- | --- |
|  | OR (95% CI) | *P* | OR (95% CI) | *P* |
| Testosterone (nmol/L) | 0.65 (0.32, 1.31) | 0.229 | 1.12 (0.62, 2.05) | 0.70 |
| T/TG ratio | 1.04 (0.73, 1.48) | 0.828 | 1.00 (0.69, 1.44) | 0.98 |
| TG (mmol/L) | 1.11 (1.06, 1.17) | < 0.001 | 1.08 (1.03, 1.14) | 0.003 |
| TC (mmol/L) | 1.15 (1.07, 1.24) | < 0.001 | 1.09 (1.00, 1.19) | 0.06 |
| HDL-C (mmol/L) | 0.60 (0.48, 0.77) | < 0.001 | 0.96 (0.71, 1.30) | 0.78 |
| LDL-C (mmol/L) | 1.14 (1.05, 1.25) | 0.003 | 0.99 (0.89, 1.10) | 0.87 |
| TC/HDL-C ratio | 1.32 (1.22, 1.43) | < 0.001 | 1.08 (0.98, 1.19) | 0.10 |
| T/TG: testosterone-to-triglycerides, TG: triglycerides, TC: total cholesterol, HDL-C: high density lipoprotein-cholesterol, LDL-C: low density lipoprotein-cholesterol, TC/HDL-C: total cholesterol-to-high density lipoprotein-cholesterol.  ^a^ Data were analyzed by model 3 adjusted for age, BMI, education level, marital status, family income, smoking, drinking alcohol, sleeping, physical activity, cardiovascular disease, hypertension, diabetes, and CRP levels. | | | | |
